# Interpretation-Based Violation Witness Validation for C: NITWIT

**DOI:** 10.1007/978-3-030-45190-5_3

**Published:** 2020-03-13

**Authors:** Jan Švejda, Philipp Berger, Joost-Pieter Katoen

**Affiliations:** 8 Johannes Kepler University, Linz, Austria; 9 University of Birmingham, Birmingham, UK; RWTH Aachen University, Aachen, Germany

## Abstract

As software verification is gaining traction in academia and industry the number and complexity of verification tools is growing constantly. This initiated research and interest into exchangeable verification witnesses as well as tools for automated witness validation. Initial witness validators used model checkers that were amended to benefit from guidance information provided by the witness. This approach comes with substantial overhead. Second-generation execution-based validators traded speed for reduced strength in case of incomplete and non-exact witnesses. This was done by extracting test harnesses and compiling them with the original program. We present the nitwit tool, a new interpretation-based violation witness validator for C programs that is trimmed to be fast and memory efficient. It verifies a record number of witnesses of SV-COMP’20 in the ReachSafety category. Our novel tool exchanges initial compilation overhead and optimized execution for rapid startup performance. nitwit borrows C semantics from the compiler used for compilation. This offloads this hard-to-get-right task and enables using several compilers in parallel to inspect possible semantic differences.

### 1 Introduction

*The importance of witnesses.* Model checking is a very successful automated verification technique with many applications. Its usage is rapidly increasing and one may fairly argue that model checking has penetrated various industries. This is true as well for software model checkers that, as opposed to first generation model checkers, directly verify program code. Model checking is in particular a very effective *bug hunting technique*: in case a property is violated, a counterexample is provided witnessing the property's violation. This is why they are often named witnesses. As phrased by Clarke *et al.* [16] "It is impossible to overestimate the importance of the counterexample feature. The counterexamples are invaluable in debugging complex systems. Some people use model checking just for this feature."

*Witness validation.* Early model checkers provided witnesses for safety properties such as "certain bad states should always be avoided" as finite paths that end in a bad state. A simple witness-steered simulation could reveal the flaw. Modern model checkers heavily use abstraction, and witnesses are no longer concrete, but rather phrased in terms of some abstract model. This is in particular true for software model checkers. Witnesses are in fact finite paths through an abstracted program representing sets of paths in the concrete program that is to be verified. These sets may contain spurious concrete paths. This raises the question whether witnesses are correct. Witness validation is the process of checking whether a witness produced by a software model checker is indeed a witness showing that the concrete program violates the property. Software model checkers such as CBMC, CPAchecker and so on, that generate witnesses are called *producers*, while software tools that perform the witness validation are named *validators*. With a single exception [12], existing validators are incorporated or directly built on top of the existing software model checkers CPAchecker [13] or Ultimate Automizer [19,18,17].

*A format for witnesses.* In order to facilitate the validation of witnesses by various different tools, a witness format has been developed that nowadays is used by many software model checkers. For safety properties as above, this format prescribes how to represent a witness for reaching a bad state. Due to this format, witnesses are exchangeable and witness validation can be done using different techniques and tools. This format allows (i) a cross-platform exchange of information that enables "drop-in" replacement of tools such as visualization and reviews of results [10], (ii) validation of witnesses which strengthens trust in verification results, especially if the verifier and validator use different techniques and (iii) a significant amount of false bug alarms to be caught by failed validation.

*Witness validation in software verification competitions.* Since a few years, the use of witnesses has become an important part in software competitions such as the annual TACAS Competition on Software Verification (SV-COMP) [2,3,4,5,6]. SV-COMP is a competition in automatic software verification, in which academic, but also some industrial, software verifiers participate. In the 2019 edition [6], 31 verifiers participated in verifying 10 522 verification tasks for C programs (and 368 for Java programs). SV-COMP has different categories, such as reachability, memory and concurrency safety, absence of overflows, and termination. SV-COMP adopted violation witnesses as part of its benchmark scoring schema since 2015 [3] and adhered to it also in the following editions [4,5,6]. This means that a verifier does not receive a point for a violated property unless the produced violation witness could be validated by at least one validator. This applies to all categories. To reflect that violation witnesses contain sufficient information for validation, the validators are granted only limited resources (e.g., only 10% of the amount of time available for verification, and 7 GB memory). Correctness witnesses were incorporated into the score evaluation in 2017 [5] – since this competition, validated correctness witnesses yield a bonus point for the producer.

*Contributions of this paper.* This paper presents the interpretation-based witness validator NITWIT. It validates violation witnesses for safety reachability properties as above. It does so for C programs. In contrast to most other validators (a) it does not rely on an existing software model checker, and (b) exploits an interpretation-based approach. NITWIT uses a home-made extension of the *PicoC* interpreter which feeds a witness automaton with steering information during a step-by-step interpretation of the C program, see Figure 1. NITWIT was evaluated on 11 533 violation witnesses in the ReachSafety category during SV-COMP 2020 and we compared its outcomes to another five witness validators that participated. NITWIT was able to validate more witnesses in this category (8 526 in total) than all its competitors, and did so substantially faster. In addition, NITWIT was able to validate 399 witnesses that could not be validated with any of the five competitors.

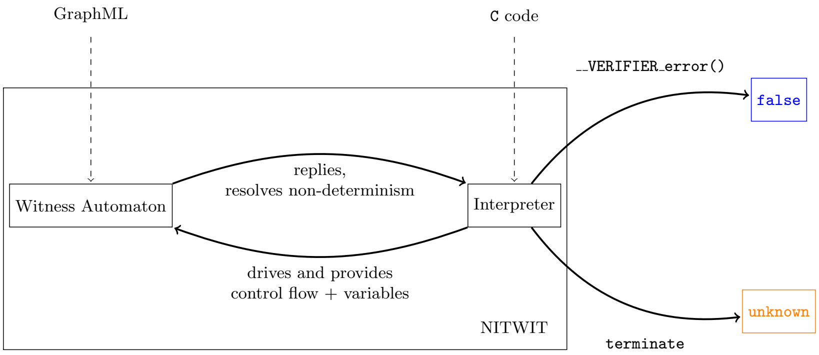

Fig. 1: High-level architecture of the NITWIT Validator.

### 2 Background

The need for achieving portability of counterexamples and proofs between tools gave rise to a type of non-deterministic finite automaton (NFA) called a *witness automaton*, or simply a witness [11]. Two types of witnesses exist – a violation and a correctness witness. In this paper, we focus on *violation witnesses*.

The concepts defined in this section follow the definitions of [22,11]. We represent programs by control-flow graphs (CFGs).

**Definition 1 (Control-flow graph).** *A* control-flow graph $\mathcal{C} = (L, l_0, G, V)$ *is a finite set of locations* $L$*, initial location* $l_0 \in L$*,* $G \subseteq L \times \mathrm{Op} \times L$ *a set of edges where* $\mathrm{Op} = \{\mathit{skip}, \mathit{assume}(\varphi), \mathit{assign}(x, E)\}$ *with* $x \in V, \varphi$ *a predicate over the program variables* $V$ *and* $E$ *an expression over* $V$*.*

In a CFG over $V = \{x, y\}$, e.g., an assignment is of the form $x := x + y$. The interpretation of a CFG is given by a (possibly countably infinite) transition system where states are of the form $(l, v)$ where $l \in L$ and $v$ is a variable assignment over $V$. For the sake of brevity, we refrain from a formal definition.

For predicate $\varphi$ over $V$, let $v \models \varphi$ denote that $\varphi$ holds in valuation $v$. A witness automaton (WA) is a finite-state automaton (NFA) used by the validator to run in parallel to the CFG such that a program run violating the specification is accepted.

**Definition 2 (Witness automaton).** *A* witness automaton *(WA)* $\mathcal{A} = (Q, \Sigma, \delta, q_0, q_E)$ *for a CFG* $\mathcal{C} = (L, l_0, G, V)$ *is an NFA with states* $Q$*, initial state* $q_0 \in Q$ *and* $\delta : Q \times \Sigma \rightarrow 2^Q$ *as usual,* $q_E$ *the accepting state and* $\Sigma \subseteq 2^G \times \Phi$*, where* $\Phi$ *is the set of predicates over* $V$*.*

The transitions of $\mathcal{A}$ have source code and guards [11] that identify program edges and place constraints on variable assignments respectively. They correspond to pairs $(D_i, \varphi_i)$, where $D_i \subseteq G$ and $\varphi_i$ is a predicate over variables.

**Definition 3 (Simulation).** *Let* $\mathcal{A} = (Q, \Sigma, \delta, q_0, q_E)$ *be a WA for a CFG* $\mathcal{C} = (L, l_0, G, V)$ *and* $\rho = l_0 \xrightarrow{g_1} \dots \xrightarrow{g_n} l_n$ *a path in* $\mathcal{C}$*. The run* $q_0 \xrightarrow{\sigma_1} \dots \xrightarrow{\sigma_n} q_n$ *in* $\mathcal{A}$ simulates $\rho$ *iff* $\sigma_{i+1} = (D_{i+1}, \varphi_{i+1})$ *with* $(l_i, g_{i+1}, l_{i+1}) \in D_{i+1}$ *and* $v_{i+1} \models \varphi_{i+1}$ *for some state* $(l_{i+1}, v_{i+1})$*. The run is* accepted *if* $q_n = q_E$ *and* $\mathcal{L}(\mathcal{A})$ *is the set of words* $\sigma_1 \dots \sigma_n$ *for which* $\mathcal{A}$ *has an accepting run.*

The path $l_0 \xrightarrow{g_1} \dots \xrightarrow{g_n} l_n$ represents a set of concrete program executions $(l_0, v_0) \rightarrow \dots (l_n, v_n)$ in which variable $x$ has value $v_i(x)$. The state conditions $\varphi_{i+1}$ restrict the set of concrete program executions to those for which $v_{i+1} \models \varphi_{i+1}$, for all $i < n$. Thus, a predicate $\varphi_{i+1}$ constrains the concrete values in $\mathcal{C}$.

When a verifier checks a property, its output should not only be *yes* or *no*, but preferably also a program execution that leads to the property violation. It is not always easy to construct a precise program execution path, as various verification techniques apply abstractions. This is taken into consideration in the witness format, for they represent a part of the state space that contains a property violation. The "narrower" the space they represent is, the easier it is to re-verify that a property is truly violated. A trivial witness automaton, e.g., which consists of only an (accepting) state with a self-loop, does not restrict the program's execution at all. Witness validation essentially then requires a verification from scratch. On the other hand, a precise witness permits only program executions leading to an error state, thereby making the validation as direct as possible.

**Definition 4 (Exact Witness).** *Let* $\mathcal{A} = (Q, \Sigma, \delta, q_0, q_E)$ *be a WA for a CFG* $\mathcal{C} = (L, l_0, G, V)$ *and* $L_E \subseteq L$ *be a set of error locations. A WA* $\mathcal{A}$ *is* exact *iff for all* $(D_1, \varphi_1) \dots (D_n, \varphi_n) \in \mathcal{L}(\mathcal{A})$ *it holds for all path* $l_0 \xrightarrow{g_1} \dots \xrightarrow{g_n} l_n$ *of* $\mathcal{C}$*: if* $(l_i, g_{i+1}, l_{i+1}) \in D_{i+1}$ *and* $v_{i+1} \models \varphi_{i+1}$ *in state* $(l_{i+1}, v_{i+1})$ *for all* $0 \leq i < n$*, then* $l_n \in L_E$*.*

### 3 Validators for Violation Witnesses

Apart from a new format for exchanging verification results, [11] also presents a feasibility study with implementing both a witness producer and a validator in two well-established tools – CPAchecker and Ultimate Automizer.

Subsequently, [12] reports on two more validators that extract test harnesses from violation witnesses to perform validation. A test harness is compiled with the program to supply input values during runtime and provide definitions for necessary external functions. This approach differs from tools using formal verification/model-checking techniques by offloading semantics to a compiler and only investigating a single path through the program. Validators that explore a single path through compilation/execution are called *execution-based* validators. In addition, a new validator MetaVal[1] was introduced in SV-COMP 2020 – we refrain from describing it as it is yet to be published though we do include it in the benchmark evaluation. All five validators participated in SV-COMP.

**CPAchecker** This tool employs a so-called Configurable Program Analysis (CPA), which allows selecting the desired level of precision to control the trade-off between performance gain and spurious counterexamples [13]. When witness validation is enabled, it matches a witness automaton against the program's CFG. Afterwards, as part of the CPA, it strengthens the exploration with state-space guards from the witness at matched locations. [11] reports that e.g. their value analysis and predicate analysis are capable of using this strengthening [15,14].

**Ultimate Automizer** This tool uses an automata-based approach to verification [19,18,17]. Prior to the analysis, it transforms programs into a variant of CFGs over an alphabet of program statements. Such a CFG, say $\mathcal{C}_{error}$, recognizes control-flow traces – sequences of statements – that lead to a property violation. A control-flow trace is *feasible* if it is a run of $\mathcal{C}_{error}$ and ends in an accepting error state. For validation, the tool creates a new CFG $\mathcal{C}_w$ from the Cartesian product of the $\mathcal{C}_{error}$ and a witness automaton. Subsequently, the tool runs the same analysis over the CFG $\mathcal{C}_w$ as for a usual verification run and validates the witness if an error trace is found. State-space guards, such as $\varphi_{i+1}$ in Definition 3 over control edges and source code guards that characterize branching are ignored.

**CPA-witness2test** This tool exploits the verifier of CPAchecker. It constructs and matches a CFG with the witness, but does not perform a CPA analysis. It collects the input and initialization values from matched assumptions and assembles an ordered vector of values for every used nondeterministic function, which it then transforms into a `switch` statement supplied as function implementation. For uninitialized variables, which in C are also nondeterministic, no values are injected.

In automatic software verification, programs are usually decorated with an external function `__VERIFIER_error` to identify a point which should never be reached, i.e., an error location. CPA-witness2test implements the function as a call to `exit(107)`, which immediately terminates an execution with return

[1] https://gitlab.com/sosy-lab/software/metaval code 107. This signals the successful validation of a witness, because the error was reached.

**FShell-witness2test** This tool does not rely on an existing software model checker. It begins with reading the specification and parses the program with `pycparser`[2] – a Python library for C, which constructs an abstract syntax tree (AST). This AST is traversed to find uninitialized variables and uses of non-deterministic functions. This yields *watch points*, indicating where variable(s) need to be resolved in order to find the right concrete path. Once watch points are established, the tool reads the provided witness and obtains a sequence of control states from program start to the error state. Further on, states of the sequence are matched to the found watch points. For any such match, the tool tries to determine the watch point value from a corresponding assumption in the witness. Finally, these values are added to a test vector, which is transformed into a test harness prepared for compilation. If the function `__VERIFIER_error` is called during execution, then the witness is accepted.

### 4 Interpretation-based Witness Validation

This section presents a new interpretation-based validator for violation witnesses of C programs with an embedded[3] reachability safety property. The validator is named Nitwit Validator (or nitwit for short) as a shorthand for iNterpretation-based vIolaTion WITness Validator. The programs *must* designate the error location by a function call to `__VERIFIER_error` in order for nitwit to recognize that a program violates the invariant "begin in `main` and never call `__VERIFIER_error`". nitwit is restricted to these programs.

*A bird's eye view on* nitwit. Our implementation approach consists of combining an existing C interpreter with a witness automaton that provides witness assumptions used for resolving variables according to the current position $(l_i, v_i)$ in the program execution. The WA is fed with information from the interpreter, which executes the C program step by step. For validations both source code and state-space guards are taken into account. When a state-space guard (an assumption) does not hold for the current variable values, then the WA does not proceed. To illustrate, suppose an integer variable $x$ initiated to one and incremented on every line (numbered from one). A witness control edge consisting of an assumption $x = 7$ matches only on line seven and will block the WA until then if no other edge is satisfied. If, however, the assumption concerns nondeterministic variables, then we extract a value from it and resolve the nondeterminism in the interpreter. E.g., if $x$ is not initialized at all, then assumption $x = 7$ assigns it the value 7 already on line one.

[2] https://github.com/eliben/pycparser

[3] The program is enhanced with error location(s) `__VERIFIER_error`, assume statements `__VERIFIER_assume` with conditions and calls to `__VERIFIER_nondet` functions, which return nondeterministic values.

As the program executes, the WA progresses through its states until either the execution ends or the error function is called. The latter we consider a testament to the property violation, accepting the witness.

*Implementing* NITWIT. An *interpreter* is a program that takes as input a program, parses it and executes commands as part of its own runtime instead of producing machine code like a compiler. Interpreters translate programs directly into the behavior they represent; they keep track of all variable values and execute statements based on results of expressions and control flow [21,1,20].

NITWIT's input is a C program. The choice of C interpreters is limited — moreover, compiled C often widely outperforms interpreters in terms of speed, due to extensive compiler optimizations and the unavoidable overhead in parsing and program state management. Nonetheless, in a witness validation setting, when a program only needs to be executed once, the advantage of machine code speed can fade away, because compilation-based validators spend effort on optimizations and translation, which is part of validation time. Furthermore, we wanted to control the simulated program during runtime to alter variables and track the position in source code, which is difficult after compilation.

Our requirements on an interpreter in the order of relevance were: (i) an open-source license permitting free use and distribution of the source code, (ii) a moderate learning curve because of the limited time for implementation, (iii) flexibility so that we can easily modify it, (iv) good coverage of C and (v) tested with realistic C programs. We have chosen *PicoC*[4], a portable interpreter written in C with a very small code base originally built as a scripting language interpreter for unmanned aerial vehicles (UAVs). In its original form, PicoC supports the basics of ANSI C, but misses some important features like function pointers or an implementation of `const` variables. For being able to execute C99-compliant C code, which is common in the benchmarks of SV-COMP, we extended it with new functionalities, such as `goto` constructs, function pointers, the `double`, `long long` and `const` types, better parsing for numerical constants, variable shadowing, `struct` initialization and bit fields.

By using an interpreter, NITWIT has full control over the simulation of a program. For our purposes, we have supplemented PicoC with function callbacks at locations corresponding to places from which a verifier might extract control-flow edges. During execution, the interpreter returns control to our witness automaton whenever it reaches a callback. The callbacks carry all of the necessary information like the current position, variable values, presence of non-determinism or the selected branch in if-statements, loops and ternary operators.

The validator's managing component stores the witness automaton and starts the program's simulation in PicoC. It also stores the current control state in the witness and tries to progress to the error state whenever it receives a callback and the source code and state-space guards match. If a state-space guard involves a nondeterministic variable, NITWIT attempts to extract a value from the given assumption. Upon success, the value is stored in the variable management system.

[4] https://gitlab.com/zsaleeba/picoc

For the assumption evaluation we execute assumptions (recall a WA-transition may have multiple of them) as conditions in the program context and if any one of them fails, then the control edge is considered as non-matching. If an assumption resolves a nondeterministic variable (e.g. the assumption `x = 2` resolves the nondeterministic variable $x$), then we automatically accept it and store the given variable value. A variable becomes nondeterministic if it has no initialization or if it is assigned a nondeterministic value (for example from a `__VERIFIER_nondet` function). Analogously, it becomes deterministic when a deterministic value is assigned to it, e.g., as a result from an expression involving only deterministic variables and constants. Moreover, if in the assumption evaluator an assumption involving a nondeterministic variable occurs and is resolved, then the variable gets assigned the new value and is registered as being deterministic.

## 5 Evaluation

### 5.1 Benchmarks

Primarily, we have tested NITWIT on witnesses produced during SV-COMP 2019 [6], however, as data from the current edition were already available to us, we present the results attained during SV-COMP 2020. The set of all witnesses produced is available at [9]. It consists of the witnesses and index files that contain information about the witness producer, date of creation, corresponding program file and its hash value (that can be used to find the program in the SV-COMP program repository), the programming language, specification, type of witness and so on. The witnesses and programs cover a large spectrum of possible language features in a variety of applications and settings. We used the dataset of the previous edition [7] to evaluate NITWIT extensively and prepare it for competing in 2020.

During the competition NITWIT was executed only on witnesses in the category *ReachSafety* with a known specification violation as our validator targets only reachability safety violations. This amounts to a set of 11 533 violation witnesses produced by 17 different verifiers.

The witnesses were not manually reviewed to check for each if the language of the WA indeed contains a violating path. This would be a laborious task — doing it automatically is a better fit, which in fact is precisely what validators are designed for. Nevertheless, this means that we cannot claim that our or other validators are incorrect when they do not find a violation, because the witness may steer them inappropriately. As the dataset does not exclusively contain exact witnesses, some witnesses might not resolve enough nondeterminism for NITWIT to find a violation based on the selected single execution.

Witnesses show a lot of heterogeneity based on their producer. Whilst some are very detailed, like in the case of PINAKA and MAP2CHECK with approximately 23 and 13 thousand nodes on average respectively, others tend to keep the WA more succinct or even minimal. For example, tools like BRICK or DIVINE usually provide the least verbose witnesses. The average number of edges typically lies near the average number of nodes due to the fact that witness producers output automata that lead directly to the error location. Not many specify information about function *enter* and *return*. Except for VeriFuzz, Map2Check and Symbiotic, tools usually put assumptions on edges selectively, though there are also some that do not use them – DIVINE and PredatorHP. Assumptions are an important part of witness automata, they restrict the exploration of state space and potentially save the most work during validation. Nevertheless, having to check a large number of them may prove difficult. On the whole, an average witness has around 2 000 nodes, 2 200 transitions between them, 1 300 state-space guards in form of assumptions, 360 controls for branching conditions, 15 function calls and return guards. The largest witness was produced by Pinaka and contains 2.1 million nodes and transitions with assumptions on almost half of them.

#### 5.2 Evaluation Setting

The runtime was limited to 90 s, while memory was limited to 7 GB [6]. Based on recorded data and extracted results, we distinguish six different outcomes of a validator:

**False** Validator found that an error location is reachable in the program. This is the desired result, nevertheless, be aware that not all witnesses in the available dataset necessarily describe valid violation paths.
**Unknown** The validator could not find a definite answer.
**True** The validator claims the program does not reach an error location in the state-space restricted by the witness.
**Timeout** The validator exceeded the granted CPU time before reaching an answer.
**Error** An error occurred in the validator during computation (*not* in the program under inspection). Includes errors due to malformed witnesses.
**Out of memory** The validator exceeded the allowed amount of memory.

#### 5.3 Experimental Results

Figure 2 presents the results on validating 11 533 witnesses by the five violation witness validators. Note that sometimes validator names in tables or plots are abbreviated for readability. The colors discern the possible outcomes described above. The validators are sorted in ascending order by the number of `False` results (blue). nitwit and CPAchecker manage to find the most violations (8 526 and 7 642 respectively), closely followed by FShell-witness2test (7 005). CPA-witness2test is able to validate 6 104, Ultimate Automizer finds 4 393 and MetaVal 1 681.

All validators except for nitwit output `True` (green) in some cases, which means the validator rejected the witness. Ultimate Automizer rejects the majority of witnesses during validation. CPA-witness2test shows the highest ratio of `Unknown` results, whereas FShell-witness2test exhibits the largest amount of unaccepted witnesses due to malformation (`Bad witness`). MetaVal exceeds the alloted time in most cases. The results are detailed in Table 2 on page 52.

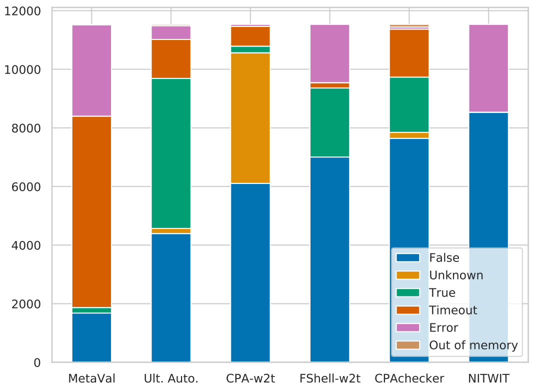

Fig. 2: Validator outcomes on 11 533 witnesses from SV-COMP 2020.

*Producing output false.* With the result `False`, validators indicate they have found a property violation, i.e., a *reachable error location*. These results are of particular interest, as the dataset used for our evaluation contains witnesses only for programs deemed incorrect.

For 10 933 witnesses at least one validator validated the verification result. Figure 3 presents a Venn diagram that displays the partitioning of these witnesses between validators based on shared successful validations. The shape as a whole stands for all of the validated witnesses and each validator is represented by a distinctly colored enclosure. Circles group intersecting results and the bigger numbers inside describe their cardinality. The smaller numbers underneath are for making clear which validators belong to the group (ordering is from top to bottom, so CPAchecker is number one and so on). The diagram reveals that only about 226 witnesses are approved by all verifiers, though the largest shared subset has 2 010 of them – it corresponds to results shared by all of the validators with exception of Ultimate Automizer and MetaVal. In total, 1 411 instances are validated only once, 1 878 twice, 2 290 thrice, 3 682 four times and 1 446 five times. nitwit validates 399 witnesses that no other tool validates. Interestingly, none of the validators subsume each other in terms of `False` results, each has some not negligible amount of witnesses validated uniquely.

Concerning resource usage, Figure 4(a) depicts the reached number of successfully validated witnesses plotted against the required CPU time (in log-scale). Data points are sorted by the required CPU time and the black line at the top marks the timeout. nitwit finds violations systematically faster than any other tool. Its mean runtime amounts to 0.63 seconds, the median was noticeably smaller at 0.02 seconds, standard deviation was 4.74. The runtime for nitwit is skewed towards zero with most results achieved under half a second. We also see that running nitwit more than 10 seconds scarcely produces any new results. That is not the case for CPAchecker, Ultimate Automizer, MetaVal and CPA-witness2test, which frequently need more than that, even though they rarely finish without a considerable headroom until the limit of 90 seconds. On average, NITWIT is about 4.2 times faster than the runner up FSHELL-WITNESS2TEST, 17.8 times than CPA-WITNESS2TEST, 22.0 times than CPACHECKER, 35.3 times than ULTIMATE AUTOMIZER and 39.1 times than METAVAL.

| **Validator** | **Result** | **Witnesses** | **Mean** | **Median** | **Std.dev.** | **Total** |
|---|---|---|---|---|---|---|
| NITWIT | False | 8526 | 0.63 | 0.02 | 4.74 | 5393 |
| | All | 11533 | 0.64 | 0.02 | 4.99 | 7386 |
| FShell-witness2test | False | 7005 | 2.67 | 1.30 | 5.90 | 18734 |
| | All | 11533 | 3.93 | 1.40 | 12.69 | 45337 |
| CPA-witness2test | False | 6104 | 11.21 | 8.40 | 8.90 | 68407 |
| | All | 11533 | 13.62 | 8.60 | 15.59 | 157037 |
| CPAchecker | False | 7642 | 13.87 | 11.00 | 11.29 | 105968 |
| | All | 11533 | 26.02 | 12.00 | 30.88 | 300145 |
| Ultimate Automizer | False | 4393 | 22.23 | 16.00 | 16.03 | 97640 |
| | All | 11533 | 28.57 | 16.00 | 28.01 | 329488 |
| MetaVal | False | 1681 | 24.66 | 17.00 | 17.81 | 41453 |
| | All | 11533 | 63.12 | 96.00 | 39.82 | 728008 |

Table 1: Runtime statistics for validators (in seconds).

Figure 4(b) shows the memory usage in successful validations plotted on a log-scale with data sorted again in ascending order. NITWIT needed the least (5 MB on average; maximum 1 GB) RAM, closely followed by FSHELL-WITNESS2TEST. The validators were only rarely approaching the limit of 7 GB (black line at the top); the largest value slightly above 4 GB during a successful validation was exhibited by CPA-WITNESS2TEST. The tools do not suffer from a lack of available memory, which is also demonstrated by the low rate of `Out of memory` results in Figure 2.

*All validations.* Figures 4(c) and 4(d) demonstrate the resource consumption of all validations. Until about the 10 500$^{\text{th}}$ witness, NITWIT remains consistently faster than all other validators, usually finishing under one second. Then, it struggles to find the answers as some witnesses do not resolve enough nondeterminism or contain very long or even infinite paths.

Compilation-based FSHELL- and CPA-WITNESS2TEST avoid the overhead of an interpreter, so are mostly able to finish before the 90 second mark, because even if the harness they extract is incomplete (still contains nondeterminism), then after compilation the execution ends quicker than if it were interpreted. In terms of absolute numbers, NITWIT takes an average 0.64 seconds per witness on the whole dataset with a median of 0.02 and standard deviation 4.99. The runtime difference on average is 3.3 seconds in favor of NITWIT compared to FSHELL-WITNESS2TEST and 13.0 seconds to CPA-WITNESS2TEST. More interesting is the median though, this was 0.02 seconds, 1.4 seconds, 8.6 seconds,

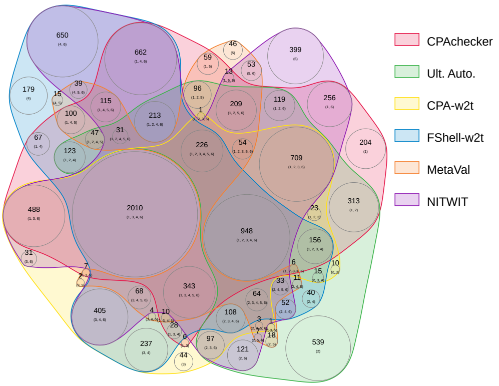

Fig. 3: A Venn diagram showing the coverage of `False` validation results by various validators.

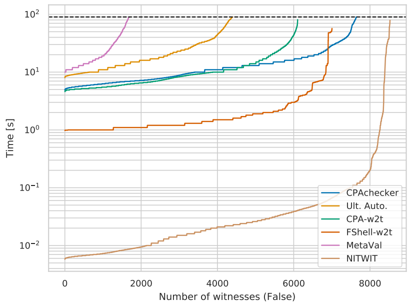

(a) CPU time (seconds) in validations for result `False`.

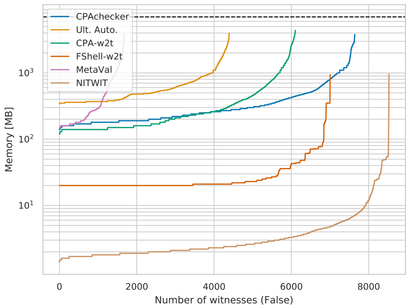

(b) Memory consumption (in MB) during validations for result `False`.

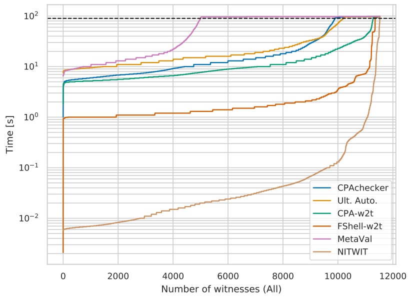

(c) CPU time (seconds) in validations for all results.

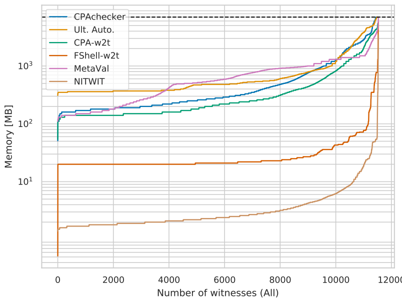

(d) Memory consumption (in MB) in validations for all results.

Fig. 4: Resources for results `False` (first row) and all together (second row).

12.0 seconds, 16.0 seconds, 96.0 seconds for Nitwit, FShell-witness2test, CPA-witness2test, CPAchecker, Ultimate Automizer and MetaVal respectively.

Figure 5 shows how nitwit compares to the other four validators in terms of time and successful validation results. In each plot, a validator is compared against nitwit. Witnesses validated by both have a blue color, validated only by nitwit yellow, by the other tool green and any other are depicted in red. The diagonal line is supplemented by two other lines representing a ±30% difference in CPU time. The result, if not `false`, is plotted on one of six lines at the end of its axis. These lines correspond to a `Timeout` (abbreviated by *to*), `Unknown` (*uk*), `True` (*tu*), `Error` (*er*) and `Out of memory` (*om*). Every point represents a witness (identical for both validators).

Figure 5 shows that in instances of agreed `False` results, nitwit is always faster than other validators. FShell-witness2test has 1 114 validations within less than one second difference. This is 0 for all of the others.

| **Verifier** | **CPAchecker** | **Ult. Auto.** | **CPA-w2t** | **FS-w2t** | **MetaVal** | **NITWIT** | **Virt. best** | **Total** |
|---|---|---|---|---|---|---|---|---|
| 2LS | 114 | 164 | 166 | 358 | 91 | 332 | 477 | 563 |
| BRICK | 20 | 11 | 38 | 36 | 17 | 38 | 42 | 43 |
| CBMC | 171 | 423 | 456 | 358 | 114 | 488 | 768 | 905 |
| CPAchecker | 1091 | 713 | 927 | 491 | 0 | 1070 | 1171 | 1189 |
| DIVINE | 400 | 46 | 110 | 280 | 181 | 237 | 448 | 460 |
| ESBMC | 572 | 131 | 605 | 843 | 249 | 730 | 955 | 1022 |
| GACAL | 0 | 10 | 0 | 10 | 7 | 15 | 15 | 15 |
| Map2Check | 80 | 32 | 106 | 129 | 120 | 137 | 211 | 264 |
| PeSCo | 1030 | 625 | 845 | 606 | 0 | 988 | 1064 | 1081 |
| Pinaka | 531 | 518 | 541 | 454 | 54 | 440 | 616 | 629 |
| PredatorHP | 55 | 35 | 18 | 44 | 53 | 20 | 69 | 70 |
| Symbiotic | 1033 | 12 | 866 | 894 | 134 | 1047 | 1103 | 1106 |
| UAutomizer | 391 | 574 | 55 | 189 | 159 | 233 | 630 | 662 |
| UKojak | 291 | 310 | 38 | 151 | 135 | 179 | 348 | 348 |
| UTaipan | 370 | 379 | 53 | 189 | 150 | 205 | 427 | 452 |
| VeriAbs | 501 | 366 | 326 | 892 | 10 | 1139 | 1298 | 1427 |
| VeriFuzz | 992 | 44 | 954 | 1081 | 207 | 1228 | 1291 | 1297 |
| Total | 7642 | 4393 | 6104 | 7005 | 1681 | 8526 | 10933 | 11533 |

Table 2: Results on successful validations of violation witnesses generated by the various verifiers. Column *Virtual best* aggregates witnesses that are validated at least once.

#### 5.4 Discussion

*Nondeterminism in programs.* nitwit is not designed for proving a program correct with respect to some specification, because the validator explores only a single path. Nevertheless, to prove a program incorrect it may suffice to look at a single path and although the program may contain nondeterministic choices (e.g., if a condition depends on a nondeterministic variable) – if these are resolved using a witness, then the execution becomes deterministic. This is the main idea behind execution- and interpretation-based validators, because after resolving

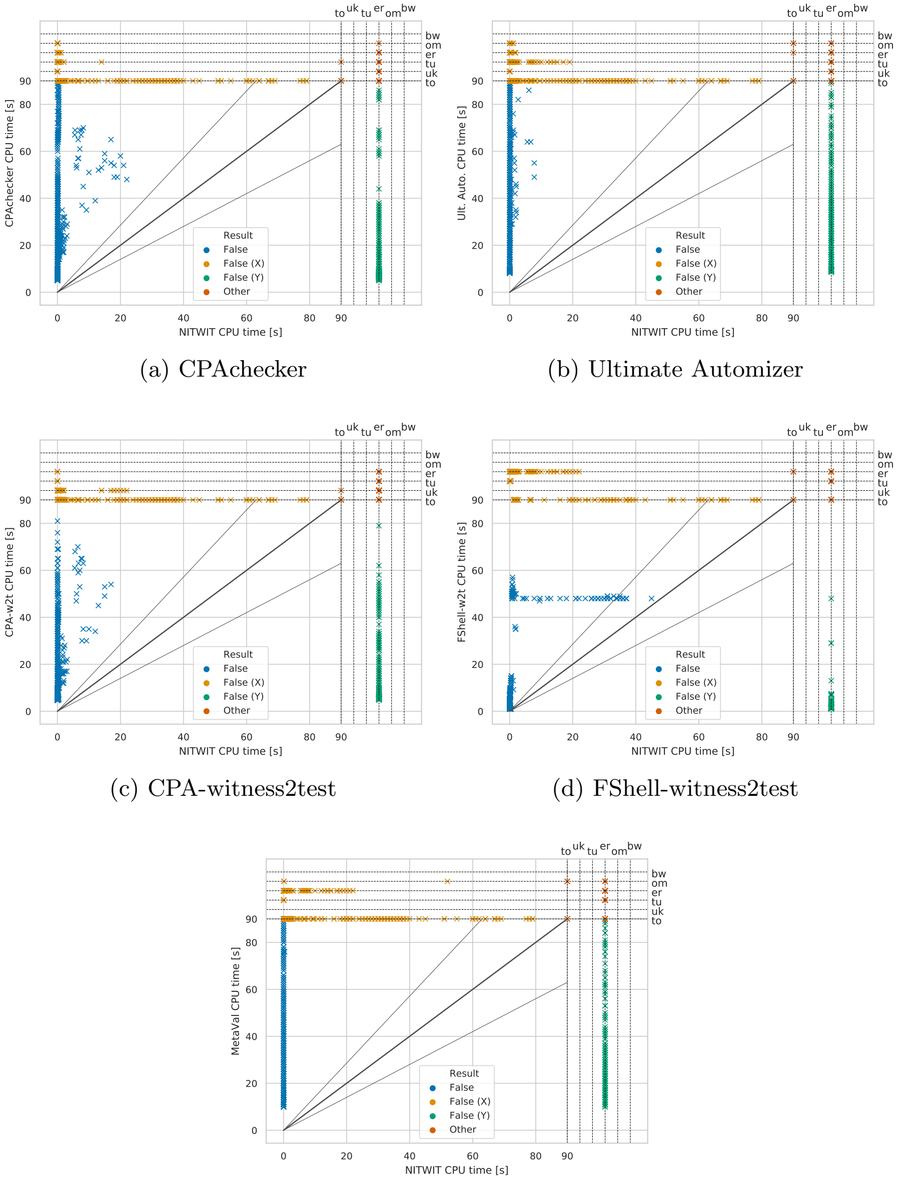

(e) MetaVal

Fig. 5: Comparing NITWIT (x-axis) with the other five validators (y-axis) in terms of speed and outcome. Each point represents a witness.

nondeterminism, there exists only a single path through the program. If this leads to an error location, then the validator may confidently claim that the provided program and witness constitute a specification violation.

NITWIT guarantees (except for implementation bugs, supported syntax and available stack- and heap size) a validated violation witness iff it allows only such abstract paths that end in an error location. Thus, given a well-specified exact witness, NITWIT should always find a violation, because it has the program state space restricted to only such paths which reach an error location. If a witness allows inexact abstract paths, then NITWIT (and in fact also an execution-based validator) may select the wrong path and see no error state. Results in Section 5 demonstrate that even without the guarantee of exact witnesses, interpretation-based validators can find a substantial amount of violations.

*Finding violations.* Results clearly show that NITWIT is a competitive validator of witnesses for C programs and invariant properties. Our validators implemented independently of any verification platform can efficiently reestablish violations from witnesses. We outperform other tools especially on the less time intensive instances as NITWIT works well in validating witnesses that restrict the state space sufficiently. For these witnesses, it is the fastest among state-of-the-art validators and has the smallest memory footprint.

We attribute the good outcomes in speed and memory to the choice of employing *an interpretation-based approach.* As NITWIT explores only one path, it is obviously faster than full fledged model-checking validators that explore many paths. Interestingly, an interpreter-based execution analysis is often much faster than compiled. This difference might be attributed to the fact that execution-based tools build the whole AST and CFG, whereas PicoC saves a lot of time by not having to construct them. Moreover, a compiler translates the program into machine code, a non-trivial task which PicoC circumvents.

*Weaknesses.* One of NITWIT's limitations is inherent to exploring only a single execution. Suppose a non-terminating program $P$, a trivial witness without assumptions and a property violation, whose reachability depends on a nondeterministic variable being zero. NITWIT, if it cannot resolve a nondeterministic variable, *assumes it has value one.* In such a setting, the simulated program diverges and so does NITWIT, because it cannot recognize an infinite execution. A similar situation may occur even if the witness is non-trivial. If its transitions are not matched to the right operations (which can be a fault in both the witness producer or validator), then $P$ will diverge due to unresolved nondeterminism.

Secondly, as we employ an interpreter, there is a noticeable overhead compared to compiled programs in terms of CPU instructions per operation. Therefore, even if an execution is finite or reaches a violation in finitely many steps, it might simply be too computationally intensive for NITWIT to provide an answer within time. Combined with unresolved nondeterminism, this explained a relatively high amount of `Timeout` results in an early version of NITWIT benchmarked on SV-COMP 2019.

To combat the timeouts, we decided to implement a simple check in the witness automaton. After a certain number of unsuccessful transitions to a different state, we deliberately stop the validation and output `Unknown`. We experimented with the threshold and concluded that 1 million attempts is appropriate. By enabling this threshold, we went from 784 to 123 killed validations and lost only 25 witnesses that would otherwise have been validated, which is an acceptable trade-off. An analysis showed that 573 of the 784 timeouts were validations of possibly non-terminating programs, 18 for terminating and the 193 remaining validations without specified termination[5]. The check for the threshold can be disabled.

*Processing witnesses.* In some cases, software verifiers do not always produce witnesses in exactly the correct format. For example, in GraphML it is necessary to define attributes for the graph, nodes and edges. If a witness happens to contain no such definitions, we supply a basic configuration that allows for its successful parsing. By default, we also do not extensively check for correctness of all of the graph attributes like the program hash.

Furthermore, we consider a reached error location as a proof of violation even if the witness automaton itself does not finish in an error state. This behavior can be changed by a compilation flag to rejection. Nevertheless, if a witness resolves enough determinism for one execution to find an error, we think it is sufficiently "good" for it to be a viable witness. For some programs, the variable resolving at the start suffices to reach a violation. However, we output a special exit code to make it clear that the witness did not in fact accept this path.

### 6 Conclusion

We presented the new interpretation-based violation witness validator NITWIT, that was able to validate 8 526 witnesses from a dataset of 11 533 witnesses [9] that were produced in the ReachSafety category of the 2020 edition of SV-COMP. NITWIT was able to validate 399 witnesses that have not been validated by any other participating tool. In addition, NITWIT has a small memory footprint and is mostly significantly faster than its competitors.

*Data Availability Statement and Acknowledgments.* NITWIT is available for free at https://github.com/moves-rwth/nitwit-validator and is licensed under the New BSD license. The replication artifact can be found at the Zenodo repository https://doi.org/10.5281/zenodo.3518139 [23] and the datasets analyzed during the current study at https://doi.org/10.5281/zenodo.3630205 [8]. We thank Dirk Beyer for very useful feedback on an earlier version of the paper and assistance with configuring NITWIT for SV-COMP 2020.

[5] We know whether these programs are (non-)terminating, as they were reviewed in SV-COMP before including them in the competition on termination analysis.
